# Ubiquitin Ligase ATL31 Functions in Leaf Senescence in Response to the Balance Between Atmospheric CO_2_ and Nitrogen Availability in Arabidopsis

**DOI:** 10.1093/pcp/pcu002

**Published:** 2014-01-30

**Authors:** Shoki Aoyama, Thais Huarancca Reyes, Lorenzo Guglielminetti, Yu Lu, Yoshie Morita, Takeo Sato, Junji Yamaguchi

**Affiliations:** ^1^Faculty of Science and Graduate School of Life Science, Hokkaido University, Kita-ku N10-W8, Sapporo, 060-0810 Japan; ^2^Department of Agriculture, Food and Environment, University of Pisa, Via Mariscoglio 34, I-56017 Pisa, Italy

**Keywords:** Biomass, C/N balance, CO_2_, Senescence, Ubiquitin ligase

## Abstract

Carbon (C) and nitrogen (N) are essential elements for metabolism, and their availability, called the C/N balance, must be tightly coordinated for optimal growth in plants. Previously, we have identified the ubiquitin ligase CNI1/ATL31 as a novel C/N regulator by screening plants grown on C/N stress medium containing excess sugar and limited N. To elucidate further the effect of C/N balance on plant growth and to determine the physiological function of ATL31, we performed C/N response analysis using an atmospheric CO_2_ manipulation system. Under conditions of elevated CO_2_ and sufficient N, plant biomass and total sugar and starch dramatically increased. In contrast, elevated CO_2_ with limited N did not increase plant biomass but promoted leaf chlorosis, with anthocyanin accumulation and increased senescence-associated gene expression. Similar results were obtained with plants grown in medium containing excess sugar and limited N, suggesting that disruption of the C/N balance affects senescence progression. In *ATL31*-overexpressing plants, promotion of senescence under disrupted CO_2_/N conditions was repressed, whereas in the loss-of-function mutant it was enhanced. The *ATL31* gene was transcriptionally up-regulated under N deficiency and in senescent leaves, and *ATL31* expression was highly correlated with *WRKY53* expression, a key regulator of senescence. Furthermore, transient protoplast analysis implicated the direct activation of *ATL31* expression by WRKY53, which was in accordance with the results of *WRKY53* overexpression experiments. Together, these results demonstrate the importance of C/N balance in leaf senescence and the involvement of ubiquitin ligase ATL31 in the process of senescence in Arabidopsis.

## Introduction

Plant growth and development are controlled by the concerted actions of signaling pathways that are triggered by various environmental conditions and developmental cues. Nutrient availability, in particular that of carbon (C) and nitrogen (N), is one of the most important factors for the regulation of plant metabolism and development. In addition to independent utilization, the ratio of C to N metabolites in the cell is also important for the regulation of plant growth, and is referred to as the ‘C/N balance’ (Coruzzi and Zhou 2001, Martin et al. 2002). In nature, C and N availability changes in response to environmental conditions, such as atmospheric CO_2_, light availability, diurnal cycles, seasonal effects, rainfall and factors influencing microbial activity (Gibon et al. 2004, Miller et al. 2007, Smith and Stitt 2007, Kiba et al. 2011). Cold and biotic stress can also affect carbohydrate partitioning and metabolism (Klotke et al. 2004, Roitsch and Gonzalez 2004). Plants sense and adapt to changing C/N conditions via precise partitioning of C and N sources and fine-tuning of complex cellular metabolic activity (Sato et al. 2011b, Sulpice et al. 2013). C/N balance clearly affects the plant phenotype in the early post-germinative growth stage. Arabidopsis seedlings grown in medium containing high levels of sugar (100 mM glucose or sucrose) and limited N (0.1 mM N) showed purple pigmentation in cotyledons and severely inhibited post-germinative growth (Martin et al. 2002, Sato et al. 2009). The expression of genes related to photosynthesis, such as *rubisco small subunit 1A* (*RBCS1A*) and *chlorophyll binding protein 2* (*CAB2*), or the anthocyanin biosynthetic enzyme *chalcone synthase* (*CHS*), is regulated by the C/N balance rather than by C or N individually. The Arabidopsis PII-like protein AtGLB1 and the glutamate receptor AtGLR1.1, which are able to bind directly 2-oxoglutarate and glutamate, respectively, are important factors in the coordinated regulation of C and N metabolism (Hsieh et al. 1998, Kang and Turano 2003, Ferrario-Mery et al. 2005). However, little is known about the molecular mechanisms responsible for the regulation of C/N sensing and signaling.

Our laboratory previously isolated the ubiquitin ligase ATL31 as a novel C/N regulatory protein in Arabidopsis plants (Sato et al. 2009). ATL31 is a member of the plant-specific ubiquitin ligase ATL family, which comprises proteins that contain a transmembrane-like hydrophobic region at the N-terminus, a basic amino acid-rich region, a RING-H2 type zinc finger domain and a non-conserved C-terminal region (Serrano et al. 2006, Aguilar-Hernandez et al. 2011). In the early post-germinative growth stage, ATL31 overexpression caused a C/N-insensitive phenotype in *carbon/nitrogen insensitive 1-dominant* (*cni1-D*) plants and resulted in the expansion of green-colored cotyledons under conditions of excess sugar and N depletion (high C/low N medium), whereas the *atl31* loss-of-function mutant showed a hypersensitive phenotype. Subsequent proteomic analyses identified 14-3-3 proteins as interactors of ATL31 (Sato et al. 2011a). 14-3-3 proteins bind to phosphorylated motifs and function in multiple developmental processes by regulating the activity of a wide variety of target proteins (Bachmann et al. 1996, Roberts 2003, Mackintosh 2004, Chevalier et al. 2009). In particular, 14-3-3 proteins have been reported to regulate primary C and N metabolism by directly interacting with essential enzymes (Comparot et al. 2003, Shin et al. 2011). Further biochemical and genetic analyses demonstrated that ATL31 targets 14-3-3 proteins for ubiquitination to regulate C/N response in Arabidopsis plants (Sato et al. 2011a). These results revealed the plant-specific regulatory mechanism of C/N nutrient signaling via the ubiquitin–proteasome system.

Supplementing exogenous sugar into the medium is a conventional and useful method for analyzing the sugar and C/N response and has revealed essential signaling factors. However, since sugar is not naturally found in soil, it is necessary to evaluate the physiological function of each signaling factor further under improved experimental conditions. The physiological C source for higher plants is sugar produced from atmospheric CO_2_. The increasing atmospheric CO_2_ concentration is a serious environmental problem as it causes elevated temperatures that could lead to major ecological consequences, such as changes in plant growth, worldwide (Long et al. 2004, Hikosaka et al. 2011, Knohl and Veldkamp 2011). Most studies have evaluated the sugar and C/N response phenotype in the early post-germinative growth stage using sugar-supplemented medium because it is a short assay and it is easy to identify differences by counting seedlings with green or purple cotyledons. However, sugar and C/N are thought to affect plant growth throughout its life cycle, including during vegetative and reproductive growth as well as during senescence (Rolland et al. 2006, Wingler et al. 2006, Watanabe et al. 2013). Here, we carried out C/N response analysis with manipulation of atmospheric CO_2_ concentrations. The combined methods of CO_2_ manipulation and hydroponic culture to regulate N levels enabled a more physiological analysis of the C/N response and the role of ATL31 in plant growth and development. Our study demonstrates that the balance between CO_2_ and N availability greatly affected not only plant biomass and carbon metabolism, but also senescence progression in rosette leaves. Both the use of sugar-supplemented medium and the manipulation of atmospheric CO_2_ levels showed that ATL31 plays a role in senescence together with the WRKY53 transcription factor. These results demonstrate the close relationship between C and N availability and the fundamental importance of the C/N balance in plant metabolism and development.

## Results

### High C and low N stress induces leaf senescence

**Fig. 1:**
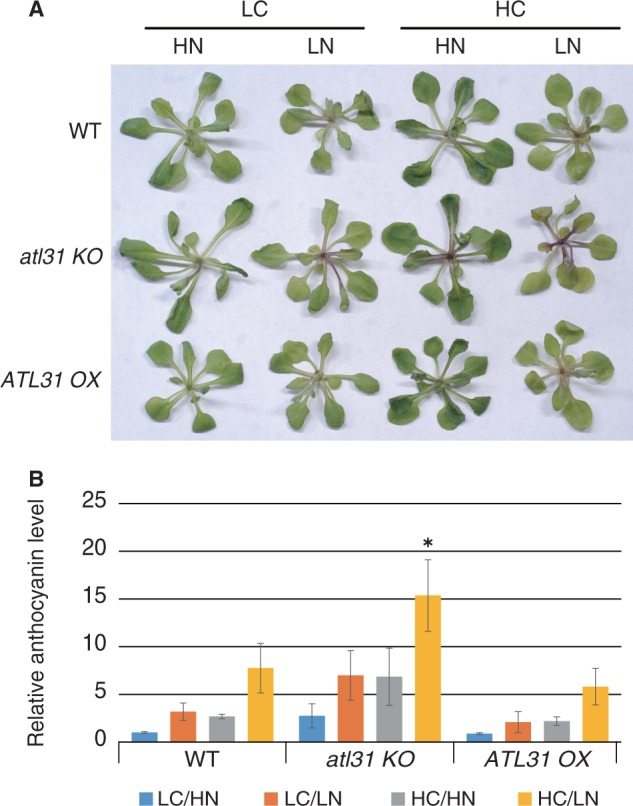
Phenotype of WT, *atl31 KO* and *ATL31 OX* plants grown in different C/N media. (A) Growth phenotype of each plant 3 d after transfer from control C/N medium (LC/HN) to modified C/N medium containing 100 or 200 mM glucose (LC or HC), and 0.3 or 30 mM nitrogen (LN or HN). (B) Anthocyanin accumulation in WT, *atl31 KO* and *ATL31 OX* plants. Anthocyanin levels in WT plants grown in LC/HN control medium was set to 1 in each condition and genotype. Means ± SD of three independent experiments are shown. An asterisk indicates significant differences compared with the WT in each C/N condition as determined by Dunnet analysis (*P* < 0.05).

**Fig. 2:**
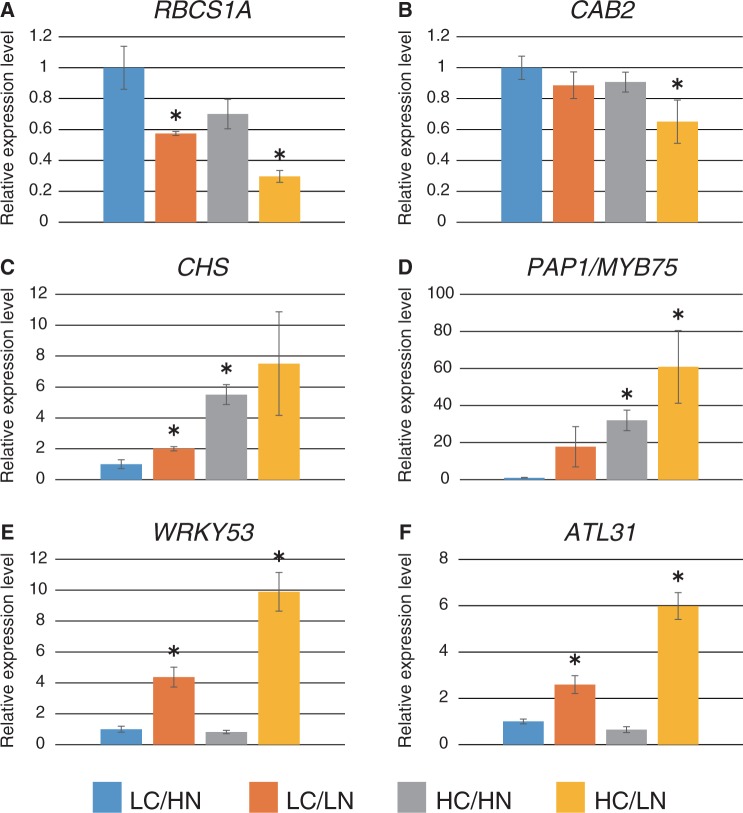
Relative expression levels of C/N- and senescence-related genes. Expression levels of C/N- and senescence-related genes in WT plants grown in each C/N medium were analyzed by qRT–PCR. Total RNA was purified from WT plants 24 h after transfer to C/N medium containing 100 or 200 mM glucose (LC or HC) and 0.3 or 30 mM nitrogen (LN or HN) from control medium (LC/HN). Relative expression levels were compared with those of WT plants grown in control C/N medium. Means ± SD of three independent experiments are shown. An asterisk indicates significant differences compared with the WT in the control C/N condition as determined by Dunnet analysis (*P* < 0.05).

In order to examine the effects of changes in the C/N ratio and *ATL31* expression in mature plants, plants were grown under different C/N conditions. Wild-type (WT) Arabidopsis plants were grown in normal C/N medium containing 100 mM glucose and 30 mM N (low C/high N) for 2 weeks and then transferred to each modified C/N medium containing 100 mM glucose and 0.3 mM N (low C/low N), 200 mM glucose and 30 mM N (high C/high N) or 200 mM glucose and 0.3 mM N (high C/low N). Three days after transfer, WT plants grown under high C/low N conditions showed slight chlorosis and increased purple pigmentation of true leaves (Fig. 1A). Anthocyanin accumulated >7-fold in high C/low N medium compared with low C/high N control medium (Fig. 1B). Plants grown under low C/low N and high C/high N conditions exhibited similar purple pigmentation (Fig. 1A), and anthocyanin accumulation increased approximately 3.2- and 2.7-fold, respectively, compared with plants grown in normal C/N medium (Fig. 1B). Gene expression of *RBCS1A*, a photosynthetic marker, decreased 0.5-fold in low C/low N and high C/high N conditions and 0.3-fold in high C/low N stress conditions compared with low C/high N medium (Fig. 2A). The expression of *CAB2* also decreased 0.6-fold in high C/low N medium (Fig. 2B). On the other hand, expression of *CHS* and *PRODUCTION OF ANTHOCYANIN PIGMENT 1* (*PAP1*)/*MYB75*, a key enzyme and a transcription factor that regulates anthocyanin biosynthesis, dramatically increased by >7- and 60-fold in high C/low N conditions, respectively (Fig. 2C, D).

Since decreased photosynthetic activity and anthocyanin accumulation are typical phenotypes of leaf senescence, expression of the senescence marker gene *WRKY53*, an essential senescence-related transcription factor (Miao et al. 2004, Lim et al. 2007), was also evaluated. *WRKY53* transcripts increased approximately 4-fold in low C/low N medium and 10-fold in high C/low N medium compared with control medium (Fig. 2E). These results suggest that C/N affects plant growth in the vegetative growth stage, and that high C/low N conditions may induce leaf senescence.

In addition, the physiological function of the C/N response regulator ATL31 was also evaluated in the mature developmental stage. Gene expression of *ATL31* was affected by C/N and increased approximately 2-fold in low C/low N medium and 6-fold in high C/low N stress conditions (Fig. 2F). Transient C/N treatments for the loss-of-function mutant [*atl31* knockout (*KO*)] demonstrated that accumulation of anthocyanin was enhanced in *atl31 KO* plants compared with WT plants grown under high C/low N conditions (Fig. 1A, B), suggesting that ATL31 is involved in C/N response at the mature growth stage. An anthocyanin accumulation was also observed in *atl31 KO* plants in response to low C/low N and high C/low N. However, there was no statistically significant difference compared with the WT (Fig. 1A, B). On the other hand, anthocyanin accumulation was not significantly repressed in *ATL31* overexpressor (*ATL31 OX*) plants compared with WT plants, although it was partially repressed in each C/N medium condition **(**Fig. 1A, B).

### CO_2_/N balance regulates plant growth and carbon metabolism

To understand further the physiological importance of the C/N balance in plants, an improved analysis of C/N response was performed using manipulated atmospheric CO_2_ concentrations as a C source. In these experiments, Arabidopsis WT plants were grown in a hydroponic culture system under differing atmospheric CO_2_ concentrations (280 or 780 p.p.m.) and N concentrations in the medium (0.3 or 3 mM).

**Fig. 3:**
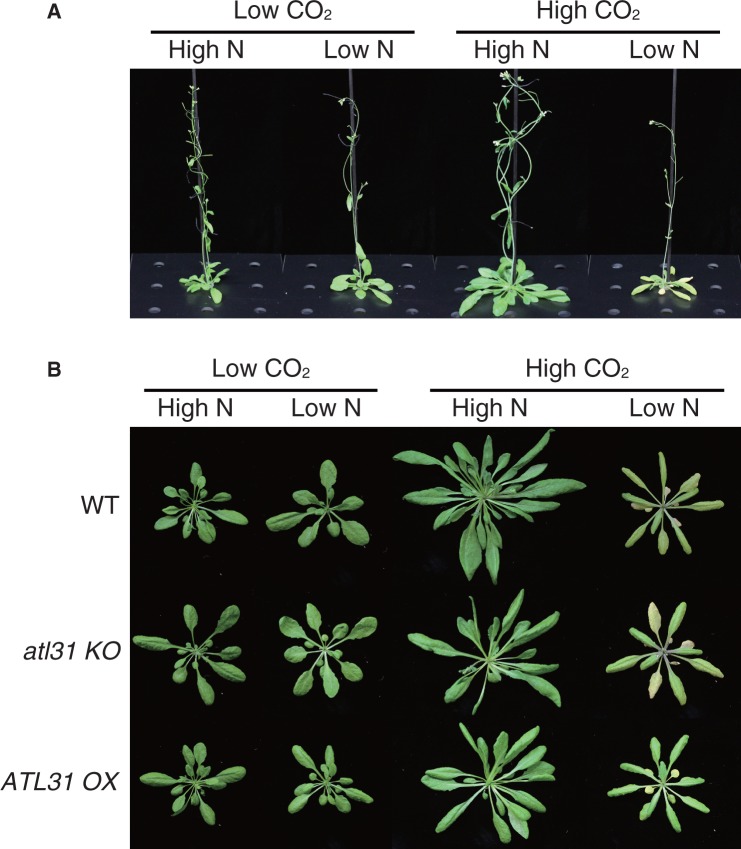
Phenotype of WT, *atl31 KO* and *ATL31 OX* plants grown under different CO_2_/N conditions. Plants were grown under 280 p.p.m. CO_2_ and 3 mM nitrogen (low CO_2_/high N) for 2 weeks and then transferred to 280 or 780 p.p.m. CO_2_ (low CO_2_ or high CO_2_) and 0.3 or 3 mM nitrogen (low N or high N) conditions and grown for an additional 4 weeks. (A) Growth of whole above-ground tissue of WT plants. The phenotypes of *atl31 KO* and *ATL31 OX* are shown in Supplementary Fig. S1. (B) Rosette leaf phenotypes of WT, *atl31 KO* and *ATL31 OX*.

**Fig. 4:**
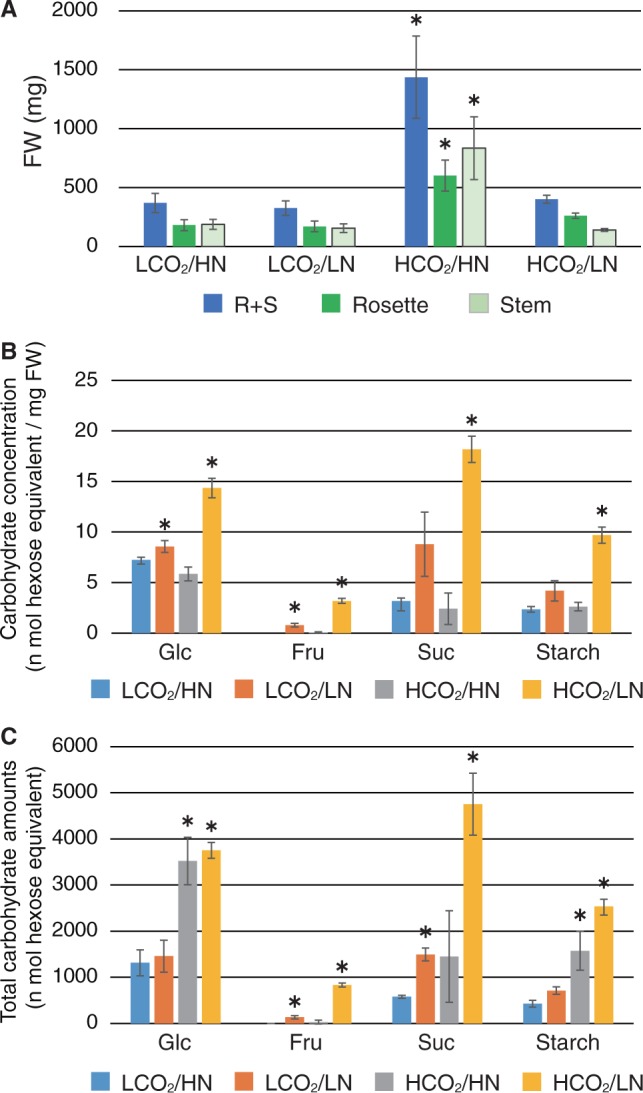
Measurement of biomass, sugar and starch amounts in response to changes in CO_2_/N conditions. Plants were grown under 280 p.p.m. CO_2_ and 3 mM nitrogen (LCO_2_/HN) for 2 weeks and then transferred to 280 or 780 p.p.m. CO_2_ (LCO_2_ or HCO_2_) and 0.3 or 3 mM nitrogen (LN or HN) conditions and grown for an additional 4 weeks. Each plant was harvested in the middle of the light period. (A) The fresh weight of WT plants grown under each CO_2_/N condition was measured. Rosette leaves (R) and stem (S) tissues were measured separately and the average of three independent experiments is shown. (B) Glucose (Glc), fructose (Fru), sucrose (Suc) and starch amounts in rosette leaves were quantified and are shown as the concentration (n mol hexose equivalent mg^–1^ FW). (C) Total amounts of carbohydrate metabolites in rosette leaves were calculated from the carbohydrate concentration and fresh weight. Means ± SD of three independent experiments are shown. An asterisk indicates significant differences compared with the WT grown under the control CO_2_/N (LCO_2_/HN) condition as determined by Dunnet analysis (*P* < 0.05).

Prior to treatment, all plants were grown under 280 p.p.m. CO_2_ and 3 mM N (low CO_2_/high N as a control) conditions for 2 weeks to avoid developmental differences between plants. Then, the plants were transferred to each CO_2_/N condition. After 4 weeks under each CO_2_/N condition, Arabidopsis plants showed apparent differences among conditions. When plants were grown under elevated CO_2_ and sufficient N (high CO_2_/high N), plant growth was dramatically promoted and leaves were enlarged (Fig. 3A, B; Supplementary Fig. S1). The fresh weight of above-ground tissues increased >3-fold compared with plants grown under low CO_2_/high N conditions (Fig. 4A). However, even with abundant CO_2_, plants grown under high CO_2_/low N conditions did not grow as much as plants grown under high CO_2_/high N conditions (Figs. 3A, B, 4A). In addition, they showed a senescence phenotype, such as purple pigmentation and chlorosis (Fig. 3B). Indeed, anthocyanin dramatically accumulated, approximately 20-fold, while Chl content decreased to less than half in plants grown under high CO_2_/low N conditions compared with those grown under low CO_2_/high N conditions (Fig. 6C, D). Since there was no visible senescence-promoting effect under either low CO_2_/low N or high CO_2_/high N conditions at the same growth stage (Fig. 3A, B), it became apparent that the senescence phenotype was not due to limited levels of N or elevated CO_2_, but was dependent upon the CO_2_/N balance.

To evaluate the effect of CO_2_/N on the biosynthesis and metabolism of C metabolites, the amounts of sugar and starch in rosette leaves were quantified. Glucose, fructose, sucrose and starch concentration (amount per fresh weight) significantly increased in plants grown under high CO_2_/low N conditions (Fig. 4B). Greater accumulation of sugars was also observed in low CO_2_/low N conditions compared with that in low CO_2_/high N, but was not greater that in high CO_2_/low N conditions (Fig. 4B). The total amount of sugars in rosette leaves in single plants was estimated by measuring the fresh weight and sugar concentration (Fig. 4C). The total glucose amount was about 3-fold higher in plants grown under high CO_2_ conditions compared with those grown under low CO_2_, indicating that the endogenous total glucose level is affected by CO_2_ but not by N conditions (Fig. 4C). On the other hand, the amount of fructose, sucrose and starch significantly increased in plants grown under low N conditions, especially when combined with exposure to high atmospheric CO_2_ levels (Fig. 4C). These results indicate that the balance between CO_2_ and N availability has a great effect on plant biomass and carbohydrate metabolism, which may affect senescence progression in plants.

### Elevated CO_2_ and limited N transcriptionally down-regulate photosynthesis genes and up-regulate senescence-related genes

**Fig. 5:**
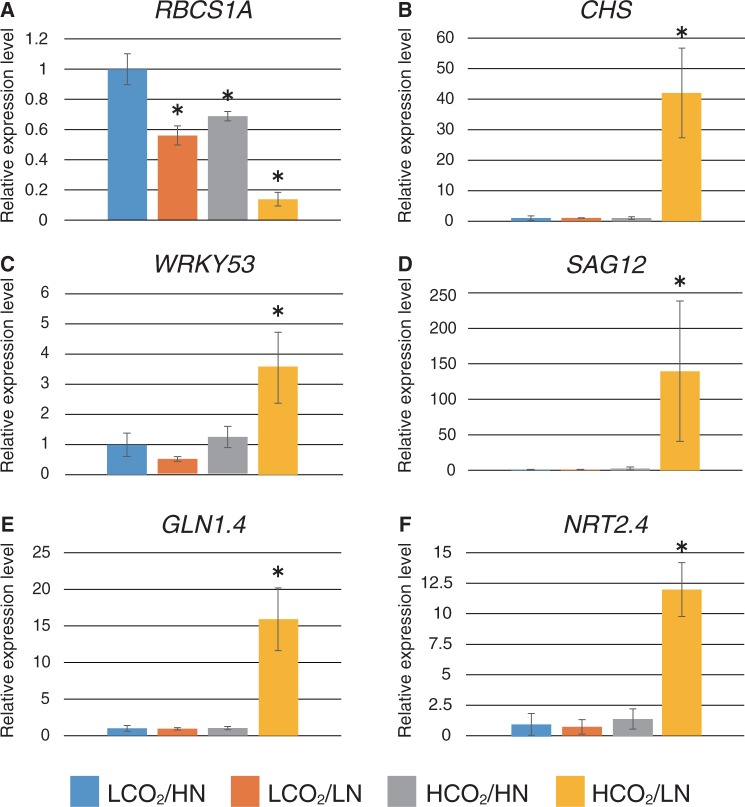
Relative expression levels of C/N- and senescence-related genes. Expression levels in WT plants grown in each CO_2_/N medium were analyzed by qRT–PCR. Total RNA was purified from WT plants grown for 2.5 weeks after transfer to each CO_2_/N condition; namely, 280 or 780 p.p.m. CO_2_ (LCO_2_ or HCO_2_) and 0.3 or 3 mM nitrogen (LN or HN). Relative expression levels were compared with WT plants grown under control CO_2_/N (LCO_2_/HN) condition. Means ± SD of three independent experiments with two technical replicates are shown. An asterisk indicates significant differences compared with the WT grown under the control CO_2_/N condition as determined by Dunnet analysis (*P* < 0.05).

Plants grown under high CO_2_/low N conditions exhibited a senescence phenotype, indicated by color changes, such as chlorosis and purple pigmentation in rosette leaves (Fig. 3B), similar to those observed in plants grown in high C/low N medium (Fig. 1A). The transcript levels of genes involved in the C/N response were examined in plants grown under each CO_2_/N condition before they showed an apparent senescence phenotype (2.5 weeks after transfer). A decrease in *RBCS1A* transcripts and an increase in *CHS* transcripts were observed in plants grown under high CO_2_/low N conditions (Fig. 5A, B) as well as in plants grown in high C/low N medium (Fig. 2A, C). These results are consistent with promotion of senescence progression. In addition, the senescence regulator gene *WRKY53* and the downstream marker *SAG12* were transcriptionally activated under high CO_2_/low N conditions (Fig. 5C, D), which is also indicative of senescence promotion.

Interestingly, the expression of cytosolic *glutamine synthase 1.4* (*GLN1.4*) and high-affinity *NITRATE TRANSPORTER 2.4* (*NRT2.4*), both of which are transcriptional markers induced by N deficiency (Ishiyama et al. 2004, Kiba et al. 2012), was also highly promoted under high CO_2_/low N conditions, although expression was not affected under low N conditions when coupled with low CO_2_ (Fig. 5E, F). These results suggest that CO_2_ and N levels affect each other and trigger senescence progression when N is depleted in plants exposed to elevated atmospheric CO_2_.

### ATL31 functions in leaf senescence under high CO_2_/low N conditions

**Fig. 6:**
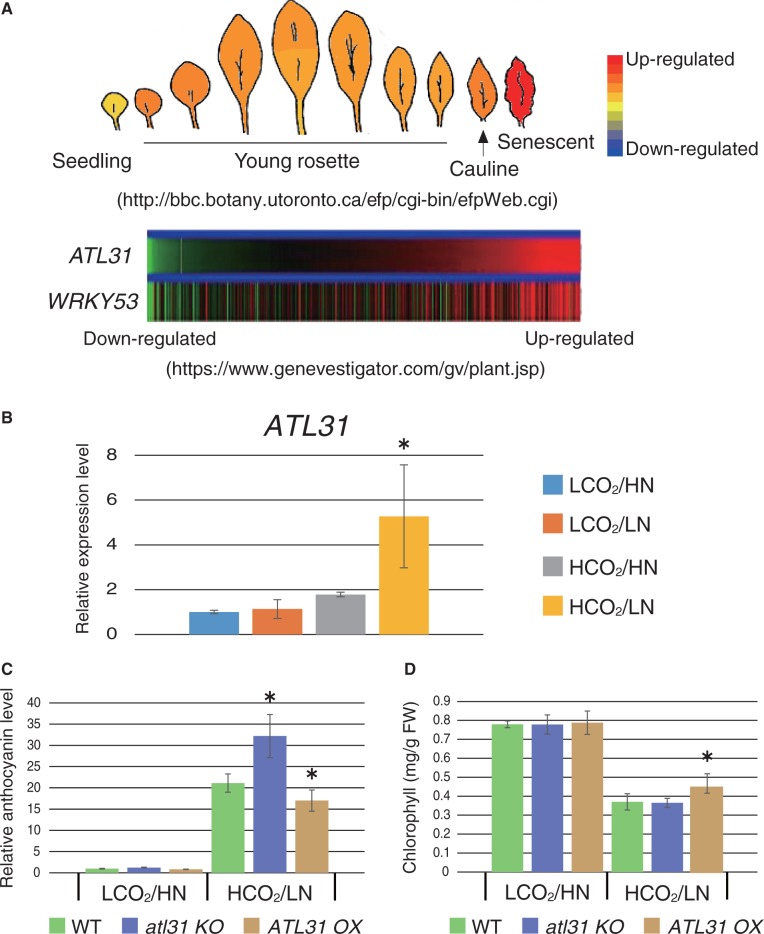
Physiological function of ATL31 in leaf senescence. (A) The expression pattern of *ATL31* is shown as researched using the publicly accessible microarray database. Age-dependent expression (eFP browser; http://bbc.botany.utoronto.ca/efp/cgi-bin/efpWeb.cgi) (upper panel) and co-expression with WRKY53 (Genevestigator; https://www.genevestigator.com/gv/index.jsp) (lower panel) were analyzed for the *ATL31* gene. (B) Expression levels of the *ATL31* gene in WT plants grown under each CO_2_/N condition were analyzed by qRT–PCR. Relative expression levels were compared with the control CO_2_/N condition (LCO_2_/HN). Means ± SD of three independent experiments with two technical replicates are shown. An asterisk indicates significant differences compared with the WT grown under the control CO_2_/N condition as determined by Dunnet analysis (*P* < 0.05). (C and D) Amounts of anthocyanin (C) and Chl (D) were quantified among WT, *atl31 KO* and *ATL31 OX* plants grown under control 280 p.p.m. CO_2_ and 3 mM N (LCO_2_/HN) or 780 p.p.m. CO_2_ and 0.3 mM N (HCO_2_/LN) conditions. Means ± SD of six independent experiments are shown. An asterisk indicates significant differences compared with the WT grown under each C/N condition as determined by Dunnet analysis (*P* < 0.05).

The physiological function of ATL31 in mature leaves was investigated under different CO_2_ and N conditions. Interestingly, it has been predicted that *ATL31* is transcriptionally induced in an age-dependent manner, which is highly correlated with *WRKY53* expression based on analysis of a publicly accessible microarray database (Fig. 6A; Supplementary Fig. S2). Promotion of *ATL31* expression in senescent leaves was also confirmed in our previous study (Maekawa et al. 2012). Quantitative reverse transcription–PCR (qRT–PCR) analysis showed that *ATL31* transcript levels increased under high CO_2_/low N conditions (Fig. 6B), which is similar to the increase seen in plants grown in high C/low N medium (Fig. 2F), suggesting an involvement of ATL31 in the progression of a senescence phenotype under high CO_2_/low N conditions.

The senescence phenotype was suppressed in *ATL31 OX* plants, whereas it was accelerated in *atl31 KO* mutants (Fig. 3B). Anthocyanin accumulation was significantly enhanced in *atl31 KO* mutants compared with WT plants grown under high CO_2_/low N conditions, whereas it was repressed in *ATL31 OX* plants (Fig. 6C). In contrast, the decrease in Chl content was suppressed in *ATL31 OX* plants as compared with WT plants and *atl31 KO* mutants (Fig. 6D). A similar accelerated senescence phenotype to the one seen in the *atl31 KO* was also observed when plants were grown under normal CO_2_ and soil conditions, whereas the *ATL31 OX* showed a slight delay in senescence progression (Supplementary Fig. S3), suggesting that ATL31 plays an essential role in leaf senescence, even under normal growth conditions. Taken together, these results demonstrate that ATL31 is transcriptionally induced in mature developed leaves under high CO_2_/low N conditions and associated with leaf senescence progression.

### ATL31 is the potential target of the senescence-related transcription factor WRKY53

**Fig. 7:**
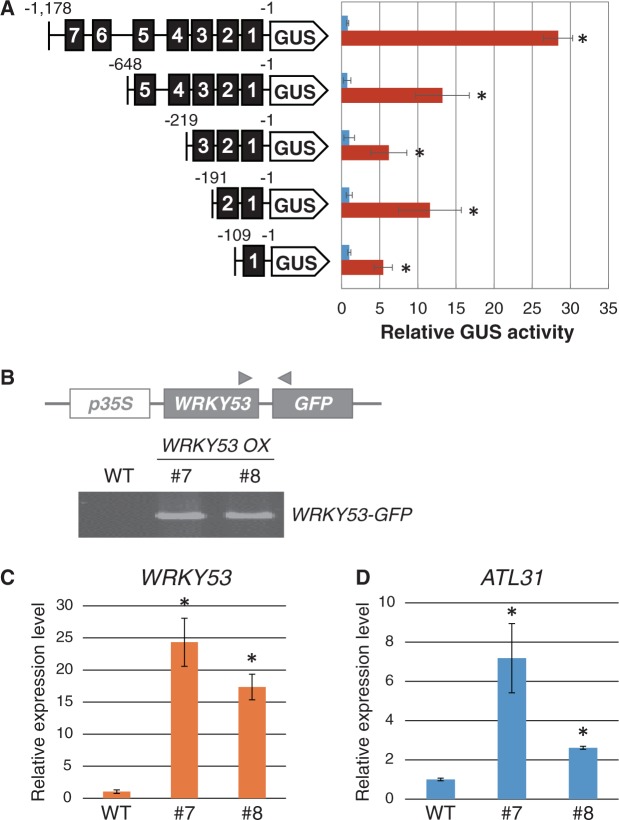
Transcriptional activation of *ATL31* by WRKY53. (A) Protoplast transient assay. Plasmids containing the promoter sequence of *ATL31* fused to the *GUS* gene and *WRKY53* coding region were co-transfected into protoplast cells, and GUS activity was measured after 15 h incubation. GUS activity was normalized to transfection efficiency. Means ± SD of relative GUS activity from three independent experiments are shown. An asterisk indicates significant differences compared with negative control cells transfected with *pATL31:GUS* and without *WRKY53* effector as determined by Student’s *t*-test (*P* < 0.05). (B) Plasmid construction and primer (arrowheads) for the isolated *WRKY53* overexpressor (*WRKY53 OX*). PCR analysis with genomic DNA confirmed isolation of *WRKY53 OX* plants. (C and D) Transcript levels of *WRKY53* and *ATL31* as determined by qRT–PCR. mRNA was purified from WT and two independent *WRKY53 OX* plants (lines 7 and 8). Relative expression levels of *WRKY53* (C) and *ATL31* (D) genes in *WRKY53 OX* plants were compared with the WT. Means ± SD of three independent experiments are shown. An asterisk indicates significant differences compared with the WT as determined by Dunnet analysis (*P* < 0.05).

As previously mentioned, *ATL31* transcription was induced during senescence, and *ATL31* expression was highly correlated with the expression of *WRKY53* (Figs. 5C, 6A, B). On the other hand, the senescence regulator WRKY53 was also transcriptionally induced in response to different C/N stress conditions (Fig. 2E). Together, these results suggest a physiologically close relationship between ATL31 and WRKY53 function. In addition, several W-box-like sequences, which are recognized as *cis*-elements by the WRKY transcription factor, were detected 5′ upstream of the *ATL31* coding region (Fig. 7A) using the *cis*-element database PLACE (http://www.dna.affrc.go.jp/PLACE/). To explore the possibility that WRKY53 directly regulates *ATL31* transcription, a reporter assay was performed using Arabidopsis protoplast cells. The *ATL31* promoter was fused to the β-glucuronidase (GUS) reporter gene (pATL31:GUS). Then, the reporter plasmid and a *Cauliflower mosaic virus* (CaMV) p35S:WRKY53 effector plasmid were co-transfected into Arabidopsis protoplast cells, followed by quantification of GUS activity. Co-transfection of WRKY53 with the *ATL31* promoter containing seven W-box-like sequences (bp −1,178 to −1) led to a 25-fold increase in GUS activity (Fig. 7A). To identify the region necessary for transcriptional activation, various deletion constructs of the *ATL31* promoter were made. Reporter analysis demonstrated that the construct containing W-boxes 1–5 (–648 to −1) was sufficient for *ATL31* transcriptional activation by WRKY53 (Fig. 7A). Additional experiments narrowed down this region even further and showed that W-box 1 (–109 to −1) was sufficient for *ATL31* induction by WRKY53 in Arabidopsis protoplast cells. It should be noted that there were significant differences in GUS activity in the absence or presence of W-boxes 6 and 7 in addition to W-boxes 1–5 (Fig. 7A), indicating that W-boxes 6 and 7 may also have some physiological effect on efficient *ATL31* induction in plants. In addition, *ATL31* transcript levels in Arabidopsis plants overexpressing *WRKY53* (*WRKY53 OX*) were analyzed. Isolation of the *WRKY53 OX* plant was confirmed by PCR with genomic DNA and by transcript analysis (Fig. 7B, C). Green fluorescent protein (GFP) fluorescence and immunoblot analysis confirmed the successful expression of WRKY53–GFP protein (Supplementary Fig. S4A, B). In *WRKY53 OX* plants, *ATL31* mRNA expression was increased compared with WT plants (Fig. 7D). These results suggest that the *ATL31* gene could be a direct transcriptional target of WRKY53 in plants.

## Discussion

### CO_2_/N affects leaf senescence progression in plants

In this study, we analyzed the C/N response upon atmospheric CO_2_ manipulation with hydroponic culturing, which revealed the effect of the C/N balance on plant growth and the close relationship between C and N metabolism. Elevated CO_2_ levels and the degree of N availability affected both plant biomass and senescence progression (Figs. 3–6). Under conditions of elevated CO_2_ and sufficient N, plants could produce more carbohydrates via photosynthesis and convert them into organic compounds such as proteins for plant growth. In contrast, under elevated CO_2_ conditions with limited N availability, plants could not grow well and instead responded with senescence progression, indicated by phenotypical changes, such as chlorosis and anthocyanin accumulation (Figs. 3B, 6C, D).

Senescence progression correlated with the increased expression of genes encoding enzymes and transporters involved in adaptation to N starvation, such as GLN1.4 and NRT2.4 (Fuentes et al. 2001, Ishiyama et al. 2004, Kiba et al. 2012) under elevated CO_2_ and limited N conditions (Fig. 5E, F). Interestingly, *GLN1.4* and *NRT2.4* expression was not up-regulated in plants grown under low CO_2_ conditions with limited N. It has been reported that plants grown under elevated atmospheric CO_2_ typically decrease cellular concentrations of N compared with plants grown under ambient CO_2_ (Coleman et al. 1993, Long et al. 2004). These results suggest that excess sugar produced under elevated CO_2_ conditions alters cellular nitrogen availability and partitioning, which disturbs the plant’s ability to coordinate N metabolism and plant growth under limited N conditions. On the other hand, N availability also affects sugar metabolism. Under limited N conditions, but not upon exposure to elevated CO_2_, the concentration of soluble sugars and starch in plants increased (Fig. 4). ADP-glucose pyrophosphorylase (AGPase), a key enzyme for starch biosynthesis, is transcriptionally up-regulated by N starvation (Scheible et al. 1997), which is also consistent with the increased accumulation of starch under elevated CO_2_ and limited N conditions observed in this study (Fig. 4B, C). In contrast, protein amounts in rosette leaves decreased approximately 0.3-fold under elevated CO_2_ and limited N compared with sufficient N conditions (Supplementary Fig. S5), together suggesting that C flux is regulated by N availability. Based on these results, we concluded that elevated CO_2_ and limited N availability mutually affect each other and thereby severely disrupt the cellular C/N balance, which leads to promotion of leaf senescence. A recent study demonstrated that a sugar metabolite, trehalose 6-phosphate (T6P), is an essential signaling molecule for the initiation of senescence in plants grown in high sugar medium (Wingler et al. 2012). Furthermore, T6P was also reported as a regulatory molecule functioning in flowering transition in Arabidopsis (Wahl et al. 2013). Thus, the role of T6P in the C/N signaling cascade should be examined in future studies.

### ATL31 functions in leaf senescence

The physiological function of the C/N-related ubiquitin ligase ATL31 was evaluated using growth media with various C/N ratios as well as combined manipulation of atmospheric CO_2_ and N availability in the medium. Gene expression of *ATL31* was induced in response to N deficiency in the C/N medium (Fig. 2F). In addition, *ATL31* expression was promoted during senescence in response to high CO_2_/low N conditions (Fig. 6B). It has been reported that the amounts of several sugars increase in the senescent leaf, whereas the amounts of nitrogen compounds decrease (Pourtau et al. 2004, Wingler et al. 2006, Wingler et al. 2012, Watanabe et al. 2013), suggesting a physiological function for ATL31 in senescence progression in response to C/N status. Actually, *atl31 KO* mutants showed a more severe senescence phenotype under elevated CO_2_ and limited N conditions, whereas *ATl31 OX* plants showed repressed senescence progression (Figs. 3, 6C, D). Similar results were also observed in the *KO* and *OX* plants grown under ambient CO_2_ and normal soil conditions (Supplementary Fig. S3). From these results, we concluded that ATL31 may be involved in senescence progression via C/N signaling and/or metabolism in plants.

It should be noted that *ATL31 OX* plants showed a different phenotype depending on whether they underwent C/N or CO_2_/N treatment. In particular, under high CO_2_/low N conditions, *ATL31 OX* plants showed a decrease in anthocyanin compared with the WT (Fig. 6C), whereas after high C/low N stress treatment *ATL31 OX* plants did not show a significant difference compared with WT plants (Fig. 1B). Previously, we have reported that *ATL31 OX* plants exhibited stress resistance and expanded green-colored cotyledons when seedlings were grown in high C/low N stress medium (300 mM glucose/0.3 mM N), whereas WT plants showed a severe growth defect with strong purple pigmentation (Sato et al. 2009). Therefore, the resistance seen in *ATL31 OX* plants may change depending on the type of C/N stress to which the plants are exposed. ATL31 is expected to contribute to the adaptation of plants to long-term limited N conditions, for instance by remobilizing nitrogen metabolites or by improving N use efficiency in mature plants.

*ATL31* gene expression is highly correlated with *WRKY53* expression. WRKY53 is a member of the large WRKY transcription factor family and positively regulates senescence-related gene expression (Miao et al. 2004, Miao and Zentgraf 2007). WRKY transcription factors are involved in diverse physiological processes, including development and secondary metabolism, as well as biotic and/or abiotic stress response (Rushton et al. 2010, Ishihama and Yoshioka 2012). WRKY proteins contain either one or two DNA-binding domains, harboring the conserved amino acid sequence WRKY, which directly binds to the W-box motif (T/CTGACC/T) in target gene promoters. Since *WRKY* genes usually contain some W-box motifs in their promoters, they can be transcriptionally autoregulated or cross-regulated by other WRKY factors. Although pull-down analysis identified several senescence-associated genes (SAGs) and other WRKY transcription factors as direct targets of WRKY53 (Miao et al. 2004), little is known about the targets, especially in regard to which targets are involved in nutrient stress-induced senescence. Results from our reporter assay in protoplasts and qRT–PCR analyses of *WRKY53 OX* plants (Fig. 7) demonstrate that WRKY53 can activate *ATL31* expression via direct binding to the W-box in the *ATL31* promoter. *WRKY53* expression was promoted not only at the senescence stage, but also in response to high C/low N conditions, similar to *ATL31* expression, implicating that WRKY53 protein physiologically controls ATL31 levels in response to the cellular C/N status in plants. Taken together, these results demonstrate that ATL31 is transcriptionally induced under high C/low N conditions and involved in senescence progression in plants. The activity of WRKY53 and other WRKY family proteins is regulated via phosphorylation by mitogen-activated protein (MAP) kinases as a part of pathogen signaling (Rushton et al. 2010; Ishihama and Yoshioka 2012). Further studies are needed to elucidate the mechanism underlying WRKY53 activation in response to C/N status, to characterize further the relationship between WRKY53 and ATL31 and to understand the upstream signaling components that regulate C/N-induced leaf senescence.

ATL31 functions as a RING-type ubiquitin ligase and regulates post-germinative growth via fine-tuning the stability of 14-3-3 proteins in response to changes in C/N status (Sato et al. 2011a). 14-3-3 proteins recognize specific amino acid motifs, including phosphorylated serine/threonine residues, and regulate many cellular signaling cascades. In particular, 14-3-3 proteins regulate C/N metabolism by directly binding to essential enzymes involved in carbohydrate and nitrogen metabolism, such as nitrate reductase, sucrose phosphate synthase, ADP-glucose pyrophosphorylase, glutamine synthetase or H^+^-ATPase (Comparot et al. 2003, Chevalier et al. 2009). To understand further the molecular mechanism underlying the regulation of primary metabolism and senescence progression in plants, we should clarify the function of 14-3-3 proteins in senescence regulation and the upstream signaling cascade modulating ATL31 activity under disrupted CO_2_/N conditions.

## Materials and Methods

### Plant materials and growth conditions

WT Arabidopsis Columbia-0 (Col-0) plants were used in this study. *atl31 KO* and *ATL31 OX* plants were prepared as previously described (Sato et al. 2009). Sterilized seeds were sown on rock wool with 1/5×MS (Murashige and Skoog) liquid medium containing 1 mM NH_4_NO_3_ and 1 mM KNO_3_ (3 mM N). Plants were grown under 280 p.p.m. of atmospheric CO_2_ concentration and 12 h light/12 h dark cycles at 22°C in a plant growth chamber for 2 weeks. Then, plants were transferred to each CO_2_/N condition; namely, 280 or 780 p.p.m. CO_2_ and 0.3 or 3 mM N. Rosette leaves for qRT–PCR analysis were harvested 2.5 weeks after CO_2_/N treatment. Rosette leaves and stem tissues for fresh weight, sugar, starch, anthocyanin and Chl measurements were harvested 4 weeks after CO_2_/N treatment.

### Transient C/N response assay

WT Col-0, *atl31KO* and *ATL31 OX* plants were grown on modified MS medium containing 100 mM glucose and 30 mM N for 2 weeks after germination and then transferred to C/N medium containing 100 or 200 mM glucose and 0.3 or 30 mM N. Plants were harvested 24 or 72 h after C/N treatment for quantitative analysis of transcript levels or anthocyanin measurements, respectively.

### qRT–PCR analysis of transcript levels

Total RNA was isolated using the RNeasy Mini Kit (Qiagen) with on-column DNase digestion according to the manufacturer’s protocol. Purified RNA (400 ng) was used for the reverse transcription reaction with Super Script II (Invitrogen). cDNA was diluted 1 : 2 with distilled water for qRT–PCR (1 : 100 dilution only for *18s rRNA*). qRT–PCR analysis was performed using SYBR premix EX Taq (TAKARA) on an Mx3000P QPCR System (Agilent Technologies) according to the manufacturer’s protocol. *18s rRNA* was used as internal control for calculating ΔΔCt. Specific primer sets used for qRT–PCR analysis are listed in Supplementary Table S1.

### Starch quantification

Leaves (0.1 g FW) were rapidly frozen in liquid nitrogen, ground to a powder and extracted twice in 0.5 ml of 80% boiling ethanol for 5 min. Samples were then centrifuged at 12,000 × *g* for 15 min at 15°C. The combined supernatants were used for soluble carbohydrate (glucose, fructose and sucrose) quantification. Pellets were resuspended in 0.5 ml of 20 mM KOH and boiled for 15 min. Samples were then centrifuged at 8,000 × *g* for 15 min at 15°C. Supernatants were utilized for starch digestion. Samples (100 µl) were combined with 100 µl of 100 mM Na-acetate (pH 5.2) containing 10 U of α-amylase (Sigma) and incubated at 37°C for 1 h. After boiling for 2 min, samples were cooled and treated with 100 µl of 100 mM Na-acetate (pH 4.6) containing 10 U of amyloglucosidase (Sigma) at 55°C for 1 h. After further boiling for 2 min, samples were cooled and centrifuged for 10 min at 15,000 × *g*. Aliquots of the supernatant were used for analysis of glucose content.

### Soluble carbohydrate quantification

Samples were assayed for glucose, fructose and sucrose content using coupled enzymatic assay methods, as described by Pompeiano et al. (2013). The efficiency of the methods was tested by using known amounts of carbohydrates. Recovery experiments determined the losses that took place during extraction procedures. Two experiments were performed for each metabolite by adding known amounts of authentic standards to the sample prior to extraction. The concentrations of standards added were similar to those estimated to be present in the tissues in preliminary experiments. The recovery ranged between 96% and 106%.

### Anthocyanin measurement

Total anthocyanin was extracted from frozen homogenized leaves by overnight incubation in 300 µl of methanol acidified with 1% HCl. Next, 200 µl of distilled water and 500 µl of chloroform were added, and the top layer was collected after centrifugation at 20,000 × *g* for 5 min and mixing with 400 µl of 60% methanol acidified with 1% HCl. The amount of anthocyanin was determined by measuring the absorbance at 532 nm (*A*_532_) and 657 nm (*A*_657_) using a spectrophotometer. Results were calculated by subtracting *A*_657_ from *A*_532_.

### Chl measurement

Chl was extracted from frozen homogenized leaves with 100% acetone. The supernatant was collected after centrifugation at 20,000 × *g* for 5 min and mixed with 1/4 the amount of distilled water. Chl content was determined by measuring absorbance at 646.6 nm (*A*_646.6_) and 663.6 nm (*A*_663.6_) as well as absorbance at 750 nm (*A*_750_) (to be used as a blank) using a spectrophotometer. Results were calculated by subtracting the blank from each absorbance. Chl *a* (µg ml^–1^) was determined using the following formula: 12.25 × *A*_663.6_ – 2.85 × *A*_646.6_. Chl *b* (µg ml^–1^) was determined using the following formula: 20.31 × *A*_663.6_ – 4.91 × *A*_646.6_.

### Plasmid construction and transgenic plant generation

To generate plasmids for transient protoplast analysis, the relevant *ATL31* promoter was amplified by PCR and introduced into the *Hin*dIII–*Bam*HI sites of pBI221 (Jefferson et al. 1987). The full-length *WRKY53* (*At4g23810*) coding region was amplified by PCR and introduced into the pENTR/D-TOPO vector (Life Technologies) to generate the plasmid pENTRWRKY53. Then, pENTRWRKY53 was recombined into the pUGW2 vector or pGWB5 binary vector (Nakagawa et al. 2007) to make an effector plasmid or GFP-fused WRKY53 plasmid, respectively, according to the Gateway instruction manual (Life Technologies), with the *WRKY53* gene under the control of the CaMV 35S promoter. Primers used for PCR amplification of promoters and effector are shown in Supplementary Table S1. The WRKY53–GFP fusion plasmid was transformed into *Agrobacterium tumefaciens* pGV3101 (pMP90) by electroporation and then transformed into *Arabidopsis thaliana* Col-0 as described previously (Sato et al. 2009).

### Protoplast transfection experiments

Protoplasts were prepared from Arabidopsis T87 suspension cells 4 d after subculture (Axelos et al. 1992) as previously described (Iwata et al. 2011). Before transfection, protoplasts were washed, centrifuged at 100 × *g* for 5 min at room temperature and resuspended to a density of 5 × 10^6^ protoplasts ml^–1^ in MaMg solution. Approximately 0.75 × 10^6^ protoplasts (150 µl suspension) were added to a mixture of 5 µg of effector and 20 µg of reporter plasmids, after which 65 µl of polyethylene glycol (PEG) solution [40% PEG (mol. wt 8,000 Da; Sigma), 0.1 M Ca(NO_3_)_2_, 0.4 M mannitol] was immediately added and carefully mixed by hand. Following incubation on ice for 20 min and at room temperature for 5 min, the transfection mixture was carefully diluted with 5 ml of wash solution. Protoplasts were pelleted by centrifugation at 100 × *g* for 5 min and resuspended in 1 ml of protoplast culture medium (0.4 M mannitol, 2% sucrose supplemented with 4.3 g l^–1^ MS basal salt mixture, 0.5 mg l^–1^ nicotinic acid, 0.5 mg l^–1^ pyridoxine hydrochloride, 0.1 mg l^–1^ thiamine hydrochloride, 2 mg l^–1^ glycine, 10 mg l^–1^ inositol, pH 5.6).

Transfected protoplasts were transferred to 3.5 cm Petri dishes, incubated under a dim light at 22°C for 15 h and lysed. The soluble extracts were divided; one half was used for analysis of reporter–GUS activity, while the other half was used for normalization. GUS activity was normalized against the relative transformed reporter–GUS plasmid amount. Protoplasts transfected with the reporter construct alone were used as control. Data are shown as the mean of three biological replicates ± SD. Predicted *cis*-elements in the ATL31 promoter were searched for using the PLACE database (http://www.dna.affrc.go.jp/PLACE/index.html) (Higo et al. 1999).

## Supplementary data

Supplementary data are available at PCP online.

## Funding

This work was supported by the Japan Society for the Promotion of Science (JSPS) Grants-in-Aid for Scientific Research (No. 24770035) to TS, on Innovation Areas (No. 24114701 and No. 25112501) to JY, and in part by The Akiyama Foundation to TS. LG was supported by the JSPS Invitation Fellowship Program for Research in Japan (n. L-13564) and SA by the Plant Global Education Project from the Nara Institute of Science and Technology (2013–2014).

## Supplementary Material

Supplementary Data

## Abbreviations

CaMV: *Cauliflower mosaic virus*
GFP: green fluorescent protein
GUS: β-glucuronidase
KO: knockout
MS: Murashige and Skoog
PEG: polyethylene glycol
OX: overexpressor
qRT–PCR: quantitative reverse transcription–PCR
T6P; trehalose 6-phosphate; WT: wild type
