# Supplementary material for: Ubiquitin Ligase ATL31 Functions in Leaf Senescence in Response to the Balance Between Atmospheric CO2 and Nitrogen Availability in Arabidopsis
Source: Plant Cell Physiol. 2014 Jan 30;55(2):293–305. doi: 10.1093/pcp/pcu002 (PMC3913444; doi:10.1093/pcp/pcu002)
Supplement: Supplementary Data [file supp_55_2_293__index.html]

Ubiquitin Ligase ATL31 Functions in Leaf Senescence in Response to the Balance Between Atmospheric CO2 and Nitrogen Availability in Arabidopsis — Supplementary Data 

# Ubiquitin Ligase ATL31 Functions in Leaf Senescence in Response to the Balance Between Atmospheric CO2 and Nitrogen Availability in Arabidopsis

## Supplementary Data

files

**Files in this Data Supplement:**

- Supplementary Data - docx file
