# Supplementary material for: Ubiquitin Ligase ATL31 Functions in Leaf Senescence in Response to the Balance Between Atmospheric CO2 and Nitrogen Availability in Arabidopsis
Source: Plant Cell Physiol. 2014 Jan 30;55(2):293–305. doi: 10.1093/pcp/pcu002 (PMC3913444; doi:10.1093/pcp/pcu002)
Supplement: Supplementary Data [file supp_pcu002_140123_Supplemental_Figure.docx]

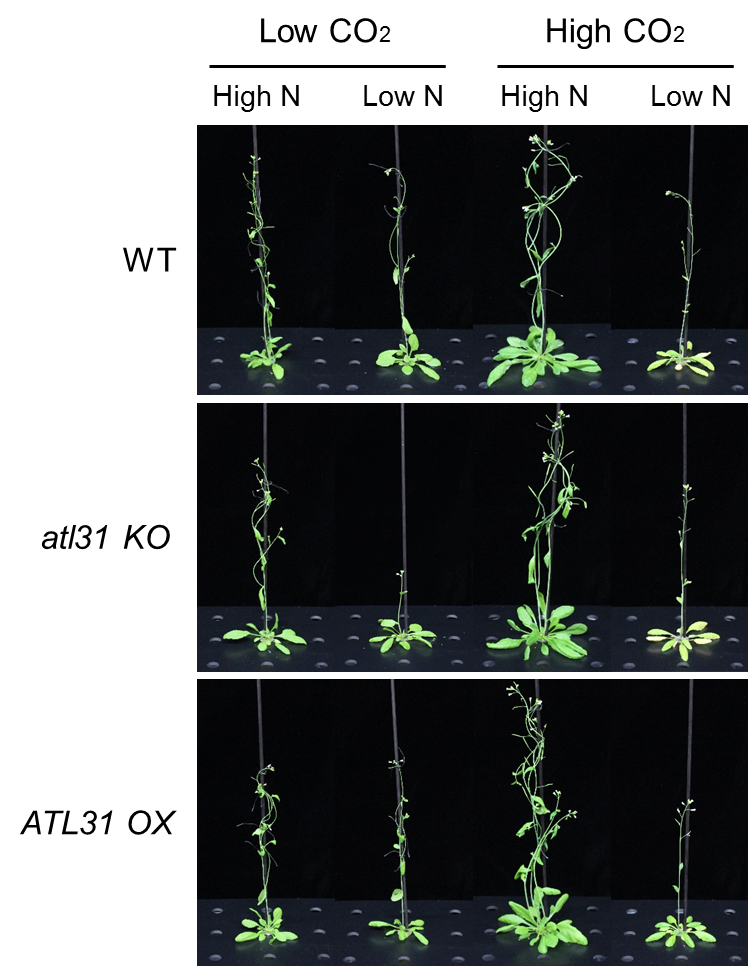


**Supplementary Fig. S1. Phenotype of WT, *atl31 KO,* and *ATL31 OX* plants grown under different CO_2_/N conditions.**

Plants were grown in 280 ppm CO_2_ and 3 mM N (low CO_2_/high N) for 2 weeks and then transferred to 280 or 780 ppm CO_2_ (low CO_2_ or high CO_2_) and 0.3 mM or 3 mM N (low N or high N) conditions. Growth phenotypes of each plant at 4 weeks after transfer to each CO_2_/N condition are shown.


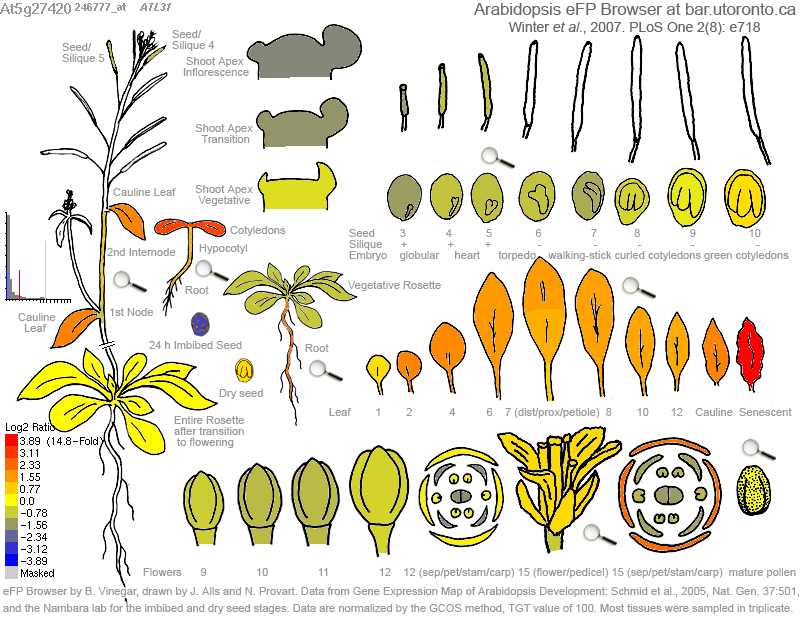


**Supplementary Fig. S2. Gene expression pattern of *ATL31* in each tissue and developmental stage.**

The expression pattern of *ATL31* was analyzed using the publicly accessible microarray database (eFP browser; http://bbc.botany.utoronto.ca/efp/cgi-bin/efpWeb.cgi). *ATL31* gene expression was strongly induced in the senescent leaf.


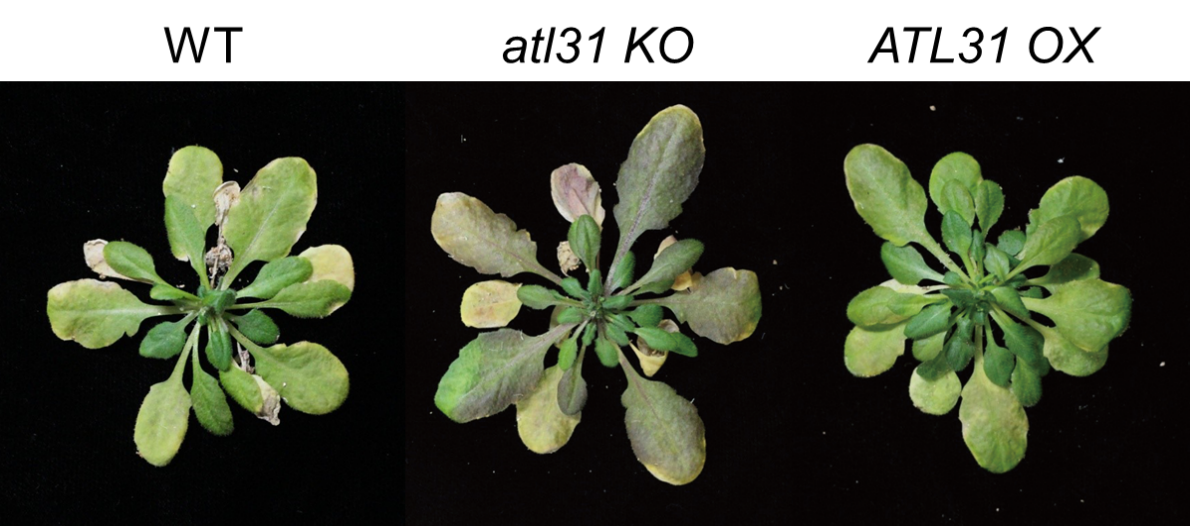


**Supplementary Fig. S3. Phenotype of WT, *atl31 KO*, and *ATL31 OX* plants.**

Plants were grown under ambient CO_2_ conditions and in normal soil containing 3 mM N. The image was taken 8 weeks after germination.

A

**
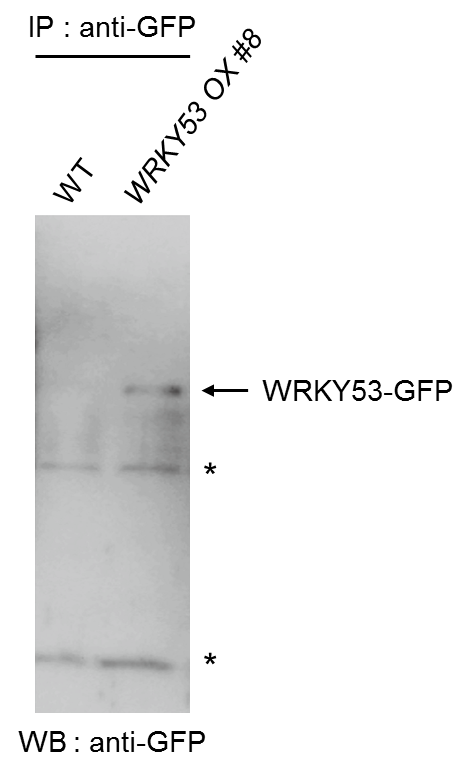
**B

**Supplementary Fig. S4. Isolation of transgenic Arabidopsis plants overexpressing WRKY53-GFP.**

(A) Observation of GFP fluorescence in the root tissue of wild-type (WT) and *WRKY53-GFP* overexpressing plants (*WRKY53 OX #8*). (B) Immunoprecipitation and immunoblot experiments using anti-GFP in WT and *WRKY53 OX #8* plants.

**Supplementary Fig. S5. Protein levels in WT Arabidopsis plants.**

Protein was extracted from the rosette leaves of WT plants grown in high CO_2_/high N or high CO_2_/low N condition for 4 weeks after transfer to each condition. Means ± SD of three independent experiments are shown. Asterisk indicates significant differences compared with WT plants grown in high CO_2_/high N condition as determined by Student’s *t-*test (*p*<0.05).

**Supplementary Table S1. List of primers used for PCR analysis.**
